# UK Dermatology Clinical Trials Network’s STOP GAP trial (a multicentre trial of prednisolone versus ciclosporin for pyoderma gangrenosum): protocol for a randomised controlled trial

**DOI:** 10.1186/1745-6215-13-51

**Published:** 2012-04-28

**Authors:** Fiona F Craig, Kim S Thomas, Eleanor J Mitchell, Hywel C Williams, John Norrie, James M Mason, Anthony D Ormerod

**Affiliations:** 1 Department of Dermatology, Aberdeen Royal Infirmary, Foresterhill, Aberdeen, AB25 2ZN, UK; 2 University of Nottingham, Centre of Evidence Based Dermatology, King’s Meadow Campus, Lenton Lane, Nottingham, NG7 2NR, UK; 3 Nottingham Clinical Trials Unit, Nottingham Health Science Partners, C Floor, South Block, Queen’s Medical Centre, Derby Road, Nottingham, NG7 2UH, UK; 4 University of Aberdeen, Centre for Healthcare Randomised Trials, Health Services Research Unit, 3 rd floor, Health Sciences Building, Foresterhill, Aberdeen, AB25 2ZD, UK; 5 Division of Applied Medicine, University of Aberdeen, Foresterhill, Aberdeen, AB25 2ZN, UK; 6 University of Durham, School of Medicine and Health, University of Durham, Queen's Campus, Wolfson Research Institute, University Boulevard, Stockton-on-Tees, TS17 6BH, UK

**Keywords:** Ciclosporin (cyclosporin), Prednisolone, Pyoderma gangrenosum, RCT

## Abstract

****Background**:**

Pyoderma gangrenosum (PG) is a rare inflammatory skin disorder characterised by painful and rapidly progressing skin ulceration. PG can be extremely difficult to treat and patients often require systemic immunosuppression. Recurrent lesions of PG are common, but the relative rarity of this condition means that there is a lack of published evidence regarding its treatment. A systematic review published in 2005 found no randomised controlled trials (RCTs) relating to the treatment of PG. Since this time, one small RCT has been published comparing infliximab to placebo, but none of the commonly used systemic treatments for PG have been formally assessed. The UK Dermatology Clinical Trials Network’s STOP GAP Trial has been designed to address this lack of trial evidence.

****Methods**:**

The objective is to assess whether oral ciclosporin is more effective than oral prednisolone for the treatment of PG. The trial design is a two-arm, observer-blind, parallel-group, randomised controlled trial comparing ciclosporin (4 mg/kg/day) to prednisolone (0.75 mg/kg/day). A total of 140 participants are to be recruited over a period of 4 years, from up to 50 hospitals in the UK and Eire. Primary outcome of velocity of healing at 6 weeks is assessed blinded to treatment allocation (using digital images of the ulcers). Secondary outcomes include: (i) time to healing; (ii) global assessment of improvement; (iii) PG inflammation assessment scale score; (iv) self-reported pain; (v) health-related quality of life; (vi) time to recurrence; (vii) treatment failures; (viii) adverse reactions to study medications; and (ix) cost effectiveness/utility. Patients with a clinical diagnosis of PG (excluding granulomatous PG); measurable ulceration (that is, not pustular PG); and patients aged over 18 years old who are able to give informed consent are included in the trial. Randomisation is by computer generated code using permuted blocks of randomly varying size, stratified by lesion size, and presence or absence of underlying systemic disease (for example, rheumatoid arthritis).

Patients who require topical therapy are asked to enter a parallel observational study (case series). If topical therapy fails and systemic therapy is required, participants are then considered for inclusion in the randomised trial.

****Trial registration**:**

Current controlled trials: ISRCTN35898459. Eudract No.2008-008291-14.

## **Background**

Pyoderma gangrenosum (PG) is a rare inflammatory dermatosis characterised by painful and rapidly progressive skin ulceration [1,2]. It most commonly affects the lower limbs, but can affect any other site including the peristomal area. It can be seen in association with a number of conditions including inflammatory bowel disease, rheumatoid arthritis, haematological disorders and malignancies [3-5]. It is a disease that causes considerable morbidity to patients, who often have a poor quality of life and high need for healthcare resources. Many patients require hospitalization for initial control, and regular changes of dressings by community care teams.

PG can be extremely difficult to treat and recurrent lesions are common [5]. There is a lack of published evidence regarding its treatment, largely because large-scale RCTs are difficult to conduct in a rare condition such as this. A systematic review in 2005 recommended the use of prednisolone, ciclosporin or high-dose intravenous steroids for large lesions; or potent topical steroids, tacrolimus or intralesional steroid injection for small lesions [6]. Other reviews have suggested a similar stepwise approach [7]. However, these recommendations were based on case series alone as no RCTs of these most commonly used treatments were identified and clinical guidelines are currently lacking. There has been one randomised controlled trial demonstrating superiority of infliximab over placebo [8], but such a potent drug would rarely be used as first line treatment. Many of the commonly used treatments are associated with unpleasant and potentially serious side effects, making their formal evaluation a matter of urgency. These treatments are currently being used for patients with PG without rigorous testing, or understanding of their relative efficacy, cost and side-effect profile.

In order to begin to address this lack of evidence, the first randomised controlled trial of systemic treatments for pyoderma gangrenosum is being carried out. The ‘Study of Treatments fOr Pyoderma GAngrenosum Patients’ (STOP GAP) will compare the two most commonly used systemic treatments: prednisolone and ciclosporin. The trial was granted ethics approval by the Northern and Yorkshire research ethics committee (MREC reference: 09/H0903/5), and all participants gave written informed consent. This paper is based on a summary of the current protocol (version 4.0, 30 August 2011. Further details are available on the STOP GAP Trial website at http:// http://www.stopgaptrial.co.uk) [9].

## **Methods**

### **Design**

**Figure 1:**
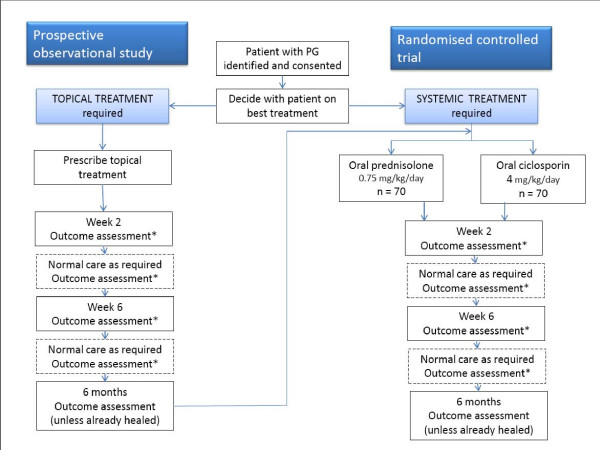
Flow chart of trial recruitment.

STOP GAP is a multicentre, parallel group, observer-blind, randomised controlled trial (Figure 1). This is a superiority trial, with prednisolone as the control intervention. The decision to power the STOP GAP trial on the basis of superiority was made as (i) ciclosporin is considerably more expensive than prednisolone (which means that it would need to be considerably better than prednisolone to warrant a change in clinical practice); (ii) case series and clinical experience give the impression that ciclosporin may gain control more quickly, and have a side-effect profile that is more suitable for long-term therapy; and (iii) pragmatically, for a rare condition such as PG, this was felt to be the best approach as an equivalence trial would require a much larger trial that could only be delivered internationally, which was beyond the scope of our funding.

The study aims to recruit 140 patients with PG over a 4-year period. Participants are randomised using a ratio of 1:1 to receive either oral prednisolone (0.75 mg/kg/day) or oral ciclosporin (4 mg/kg/day). Patients who initially require topical therapy are asked to enter a parallel observational study, the purpose of which is to provide an estimate of treatment response to topical therapy in a well defined prospective case series, and also to enhance recruitment by allowing those who fail on topical therapy to enter the main study (Figure 1).

Follow-up is continued until the lesion has healed, or for a maximum of 6 months (whichever is sooner).

## **Objectives**

The primary objective for this trial is to assess the speed of response to treatment for the compared treatments.

Secondary objectives are to assess overall treatment response (especially time to healing), safety, and cost effectiveness of the compared treatments.

### **Participants**

Patients aged over 18 years with a clinical diagnosis of PG (as diagnosed by the recruiting dermatologist) can be enrolled into the study. There must be measurable ulceration (that is, not pustular PG) for the patient to be included. In keeping with the pragmatic nature of the study, a positive skin biopsy is not required for diagnosis since histology is rarely diagnostic, but should be performed as part of normal care in cases of diagnostic uncertainty in order to exclude alternative diagnoses. For cases where there is doubt over the clinical diagnosis, an expert panel is available to provide assistance in confirming or refuting the diagnosis.

Patients are not eligible for the study if they: (i) have granulomatous PG (which is very rare, and may respond differently to treatment); (ii) have received oral prednisolone, oral ciclosporin or intravenous immunoglobulins within 1 month prior to randomisation; or (iii) are already participating in another clinical trial. In addition patients are not eligible for the RCT arm of the study if they (i) are pregnant, lactating or at risk of pregnancy; (ii) have known hypersensitivity to either of the study drugs; (iii) have a biopsy result that is consistent with a diagnosis other than PG; (iv) have clinically significant renal impairment that would result in the investigator not normally treating with either study drug; (v) have any pretreatment investigations, the results of which would prompt the investigator not to use either study drug; (vi) have any malignant of premalignant disease where treatments might interfere with ongoing therapy or might cause harm; (vii) have any concurrent medical condition which would preclude treatment with either of the study medications; (viii) administration of a live vaccine within 2 weeks prior to randomisation; or (ix) are currently taking Rosuvastatin (Crestor) for hypercholesterolaemia, since this is contraindicated in patients taking ciclosporin.

### **Interventions**

Participants are randomised to receive either oral prednisolone 0.75 mg/kg/day or ciclosporin (Neoral) 4 mg/kg/day (in two divided doses), up to a ceiling dose of 75 mg/day prednisolone and 400 mg/day ciclosporin. The dose of study drug can be adjusted (up or down) by the investigator according to response after the week 2 visit. If possible the dose of study drug should not be altered for the first 2 weeks of the study.

The use of a double dummy design was not felt to be appropriate for this study as the side effect profile, monitoring requirements and dosing regimen for the compared drugs is very different, making such a design both costly and possibly ineffective. As a result the trial team opted to ensure blinded outcome assessment of the primary outcome (using digital images) and secure allocation concealment. Participants are prescribed study medication from standard pharmacy supplies at each recruiting hospital.

Participants of the RCT should not use any topical therapy (for example, corticosteroids or calcineurin inhibitors) after randomisation. If topical therapy has been prescribed while the participant is waiting to enter the study it should be stopped when the participant enters the RCT. All other concomitant medications should continue as per normal care. The use of live vaccines is not permitted during the intervention phase of the trial and clinicians will be advised not to start Rosuvastatin (Crestor) on any patients receiving ciclosporin as it is contraindicated. Participants are asked to assess how well they adhered to study drug at week 6 and at 6 months (or time of healing).

Participants in the observational arm of the study receive whichever topical therapy is prescribed as per normal care at the recruiting hospital (this is most likely to be clobetasol propionate (Dermovate) or tacrolimus (Protopic 0.1% or 0.03%) as there is reasonable evidence to support the use of both of these treatments [10,11].

### **Randomisation and blinding (masking)**

The randomisation schedule has been created by the Nottingham Clinical Trials Unit data manager using the ralloc Strata (College Station, TX, USA) add-on. Participants are randomised to treatment allocation using a computer-generated pseudorandom list using permuted blocks of randomly varying size between 2 and 6. Randomisation is stratified by lesion size (≥20 cm^2^ vs lesions <20 cm^2^) and presence or absence of underlying systemic disease. The randomisation schedule will be concealed until interventions are all assigned and recruitment, data collection, data cleaning and analysis are complete.

This is an observer-blind study. The primary outcome (velocity of healing) is assessed on the basis of digital images of the target lesion in order to protect against detection bias. The digital images will be assessed by two assessors who are blinded to treatment allocation, using patient number as the identifier. The images will also be used to assess global improvement in disease and the PG inflammation scale (if possible, depending on the quality of the images).

Both clinicians and participants know their treatment allocation and so no special measures are required to allow for breaking of treatment codes. However, treatment allocation will not be revealed to the recruiting physician until participants’ details and key stratification variables have been irrevocably entered by the physician onto the web-based randomisation site.

### **Primary outcome**

The primary outcome is velocity of healing at 6 weeks. This will be captured for a single target lesion per patient and measured using digital photography and Verge Videometry VEV MD (Vista Medical, Winnipeg, Canada) computerised planimetry [12]. If multiple lesions are present, the target lesion should be the lesion that is most able to be photographed on a single plane (that is, not around the curvature of a limb). Digital images will be taken at baseline, 6 weeks and when the ulcer has healed (maximum 6 months). In addition, maximum longitudinal length and maximum perpendicular length will be measured in order to provide some measure of improvement in case of difficulties with the digital images, or if the lesion extends around the curvature of a limb. This will be converted to approximate area by the formula: length × width × 0.785, which approximates to an ellipse for the purpose of randomisation and analysis.

Velocity of healing at 6 weeks was chosen as the primary outcome as it is unlikely to be compromised by the single blind nature of the study, and does not require lengthy follow-up, thus minimising the possibility of missing data. Previous work in patients with venous leg ulcers suggests that velocity of healing is a good surrogate for subsequent healing [13].

### **Secondary outcomes**

Secondary outcomes include the following: (1) time to healing assessed by participants (estimated to the nearest week) based on the time at which sterile dressings are no longer required for the wound. Healing is confirmed by the clinician, and documented using digital photography at the first opportunity. Time to healing is important clinically as it will be used to confirm the primary outcome as a useful surrogate measure. It also gives an indication of duration of treatment, which therefore provides information on cumulative drug toxicity. (2) PG specific global assessment of efficacy (derived from a study by Foss *et al.*[14]) as assessed by the clinician and the patient at 2 weeks, 6 weeks and at 6 months (or healed). This will also be assessed on the digital images by an independent assessor. (3) Inflammation assessment scale. This is a combination scale including erythema, border elevation and exudate (based on a scale reported by Foss *et al.*[14]). The inflammation assessment scale is recorded by the clinician and the participant at baseline, 2 weeks, 6 weeks and when the ulcer has healed (maximum 6 months). This will also be assessed on the digital images by an independent assessor. (4) Self-reported pain. Each day for the first 6 weeks of the trial participants report the severity of the pain in a study diary (none, mild, moderate, severe or extreme), and whether or not painkillers were taken. (5) Health-related quality of life assessed at baseline, 6 weeks and 6 months (or healed), using validated questionnaires: Dermatology Life Quality Index [15] and the EQ-5day [16]. (6) Time to recurrence assessed at the end of the trial. A recurrence is defined as the occurrence of a further episode of PG (at any site) that appears after the target lesion has been confirmed as being healed by a physician (or nurse). Self-reported healing that has not been confirmed by a medical professional, and which subsequently recurs, will not be classed as a recurrence and handled as a continuation of the initial episode. (7) Number of treatment failures. Treatment failures are defined as being participants who withdraw (or are withdrawn) from their randomized treatment because of treatment intolerance or worsening of the PG, or those who continue to have any unhealed ulcers after 6 months of follow-up. (8) Adverse reactions to study medications. Adverse events that are classed as possibly, probably or definitely relating to the study medication. (9) Cost effectiveness of the compared treatments.

For participants in the observational study, only efficacy outcomes are being collected (no safety or cost-effectiveness outcomes will be reported).

### **Sample size**

A total of 140 patients (randomised 1:1 to prednisolone or ciclosporin) will give the study at least 80% power at a 5% level of significance using a two-sided two-sample *t* test to detect a difference in means of 0.5 SDs of the primary outcome of velocity of healing at 6 weeks. The velocity of healing at 6 weeks is defined as the percentage change in surface area (measured by planimetry using digital photographs) over baseline of the target lesion. This sample size allows for an approximate 10% loss to follow-up at 6 weeks. These calculations were performed using Nquery 6.0.2 on Windows XP (Microsoft, Redmond, WA, USA).

### **Statistical analysis**

The primary analysis will be according to the intention to treat principle, and will adjust for known prognostic baseline covariates. There are no formal planned interim analyses, but progress reports on all data issues are presented to a Data Monitoring Committee (DMC).

The primary outcome is to be assessed at 6 weeks, which reduces the likelihood of having missing data for this outcome. If digital images are not available for any participants, then the physical length and width measurements recorded by the clinician will be used in place of the computer generated planimetry measurements. If neither a digital image, nor physical measurements are available at 6 weeks, multiple imputation will be used using the assumption that the data are missing at random.

All primary outcome data will be double data entered and 10% of other data will be entered twice and checked for errors.

Categorical variables will be summarised with the number and proportion of participants falling in each category. Continuous variables will be summarised using the number available, number missing, mean and standard deviation (SD), or median, 25th and 75th quartiles, and minimum and maximum values as appropriate.

Differences between treatment groups for the primary and secondary outcomes will be assessed using Student’s *t* test. Linear regression models, adjusting for baseline covariates, will be used to compare the treatment groups at 6 weeks for the primary outcome, and any other continuous secondary outcomes that fit the assumptions. Cox regression models, adjusting for baseline covariates, will be used to compare the treatment groups in the analysis of the time to healing of the target lesion and the time to recurrence. Proportional odds models, adjusting for baseline covariates, will be used to analyse the categorical secondary outcomes, including global assessment of improvement and the inflammation assessment scale. The self-reported pain and the EQ-5D score will be summarised by the area under the curve (AUC), using Generalized linear models with the appropriate distribution. Patients are required to have the data recorded at the initial timepoint, and at the 6 week timepoint (for the 6 week analyses) or the final visit (for the final visit analyses), so only patients with at least the first observation and the last observation for the self-reported pain data, or those with both timepoints for the EQ-5D data, will be analysed in the first instance. Sensitivity analyses will be carried out for the self-reported pain outcome using the ‘last value carried forward’ method for patients who have missing interim data.

### **Cost effectiveness**

Costs of the compared treatments will be assessed from the perspective of the health service, using resources such as inpatient stays, outpatient attendances, primary care appointments (at surgery), home visits by GP, district nurse visits and PG-related treatment costs. Cost effectiveness will be presented as the cost per healed ulcer at 6 months.

Health related quality of life will be estimated from responses to the Dermatology Life Quality Index. Quality-adjusted life years will be assessed from responses to the EQ-5D questionnaire, and the incremental cost per quality-adjusted life years gained will be presented for the compared treatments. Health state utilities will be calculated using UK population tariffs. Estimates of resource use (from medical records and patient diaries) will be combined with unit costs, to derive total costs. Unit costs will be based on study specific estimates and data from standard sources. Point estimates of mean incremental costs, QALYs, cost per QALY and cost per resolved episode at 6 months will be reported.

### **Study organisation and funding**

The study is being coordinated through the UK Dermatology Clinical Trials Network [17] and the Nottingham Clinical Trials Unit at the University of Nottingham. It is funded by the National Institute for Health Research (NIHR) as part of an NIHR Programme Grant (RP-PG-0407-10177), and sponsored by the Nottingham University Hospitals NHS Trust. Further research nurse support is provided through the NIHR Clinical Research Networks [18].

The trial is being overseen by a Trial Steering Committee, which includes an independent chair and three other independent members (one of whom is a patient with PG). The DMC meets annually (or more frequently as required), and oversee all ethical and safety issues in accordance with current regulations and MRC guidelines for data monitoring committees. All members are independent of the study team, although the Trial Manager and some other members of the Trial Management Group (TMG) attend the open sessions in order to inform the DMC of trial progress. This committee meet at least once a year.

## **Discussion**

The STOP GAP trial is a unique study that could not be achieved without the collaborative efforts of large numbers of participating dermatologists, nurses, NHS Trusts and research networks. Recruitment is taking place at approximately 50 UK (and Ireland) secondary care dermatology departments, all of whom are contributing just 1 or 2 participants per year. Investigators are contributing on a voluntary basis because they believe that this is an important clinical question that will help to inform their clinical practice. Such a recruitment model means that it is not possible to provide dedicated STOP GAP research nurses at individual sites, and that the recruiting dermatologists are sometimes isolated. However, the development of the National Institute for Health Research (NIHR) Clinical Research infrastructure in the England (and CRC Cymru in Wales) means that support is now available to deliver collaborative trials of this kind, and many nurses are available within participating NHS Trusts to assist with patient recruitment and follow-up.

As a model for conducting trials of rare skin conditions, the STOP GAP trial is methodologically interesting in that efforts have been made to include all PG patients who are willing to take part, by inclusion in either the RCT or the observational study. This means that the trial will not only deliver the largest RCT ever to have been conducted in PG patients, but also the largest prospective case series of patients treated with topical therapy.

The use of a parallel observational study alongside the RCT also has advantages for recruitment as it means that patients with mild disease, who are initially considered to be ineligible for the main RCT, remain in contact with the trial team. If topical treatment fails and systemic therapy is indicated, these patients can then be considered for inclusion in the RCT. This approach is particularly useful for rare conditions, where few patients are seen at recruiting centres and the evidence- base for treatment is poor (making a prospective case series more valuable).

### **Trial status**

Recruitment into this trial started in May 2009 and is taking place in approximately 50 secondary care hospitals in the UK and Ireland. It is anticipated that recruitment will be completed by December 2012 at the latest.

## **Competing interests**

The authors declare that they have no competing interests.

## **Authors’ contributions**

All authors contributed to the design and writing of the full STOP GAP protocol and are members of the Trial management Group. FC and KT wrote the first draft of this paper; all authors commented and amended drafts of the paper and approved the final version. All authors read and approved the final manuscipt.
